# Preparation and Optimization of Triptolide-Loaded Solid Lipid Nanoparticles for Oral Delivery with Reduced Gastric Irritation

**DOI:** 10.3390/molecules181113340

**Published:** 2013-10-29

**Authors:** Cong Zhang, Conghui Gu, Fan Peng, Wei Liu, Jiangling Wan, Huibi Xu, Christopher Waikei Lam, Xiangliang Yang

**Affiliations:** 1 National Engineering Research Center for Nanomedicine, College of Life Science and Technology, Huazhong University of Science and Technology, Wuhan 430074, China; E-Mails: congzhang1983@gmail.com (C.Z.); guconghui@126.com (C.G.); pengfan201208@163.com (F.P.); wliu@mail.hust.edu.cn (W.L.); wanjl@mail.hust.edu.cn (J.W.); hbxu@mail.hust.edu.cn (H.X.); 2 State Key Laboratory of Quality Research in Chinese Medicine, Macau Institute for Applied Research in Medicine and Health, Macau University of Science and Technology, Macau, China

**Keywords:** triptolide, solid lipid nanoparticles, microemulsion technique, central composite design, sustained release, gastric irritation

## Abstract

Triptolide (TP) often causes adverse reactions in the gastrointestinal tract when it is administered orally. This study aimed to prepare and optimize triptolide-loaded solid lipid nanoparticles (TP-SLN) with reduced gastric irritation. The microemulsion technique was used to formulate TP-SLN employing a five-level central composite design (CCD) that was developed for exploring the optimum levels of three independent variables on particle size, encapsulation efficiency (EE) and drug loading (DL). Quadratic polynomial models were generated to predict and evaluate the three independent variables with respect to the three responses. The optimized TP-SLN was predicted to comprise fraction of lipid of 49.73%, surfactant to co-surfactant ratio of 3.25, and lipid to drug ratio of 55.27, which showed particle size of 179.8 ± 5.7 nm, EE of 56.5 ± 0.18% and DL of 1.02 ± 0.003% that were in good agreement with predicted values. In addition, the optimized nanoparticles manifested a sustained-release pattern *in vitro* and were stable during 3 h of incubation in simulated gastric fluids without significant size change and the majority (91%) of the drug was protected. Furthermore, the nanoparticles did not show obvious gastric irritation caused by oral administration of TP in rats.

## 1. Introduction

Triptolide (TP) is a major active and toxic component isolated from the traditional Chinese medicine *Tripterygium wilfordii* Hook F (TWHF). TP has multiple biological activities, including anti-inflammatory, immunosuppressive, anti-fertility, anti-cystogenesis and anticancer activities, however, the clinical utility of TP has been limited by its poor water solubility and high toxicity [1,2,3]. The most common adverse reactions to TP often occur in the gastrointestinal tract, such as nausea, anorexia, vomiting, diarrhea, gastrointestinal ulcer and bleeding [1,4,5]. However, the oral route is the simplest and most preferred route for administration of drugs as it offers the greatest degree of patient compliance [6,7]. Therefore, development of novel delivery systems of TP would be useful for minimizing gastrointestinal irritation.

Solid lipid nanoparticles (SLN) were introduced at the beginning of the 1990s as a new colloidal drug delivery system with advantages such as nontoxicity, excellent biocompatibility, and large scale production facilities, which made SLN interesting alternatives to liposomes, microemulsions, and other polymeric nanoparticles [8,9,10]. Due to their solid matrix, solid lipid nanoparticles can protect the incorporated drug from chemical degradation in the gastrointestinal environment and have been extensively investigated as a promising drug delivery system for controlling the release of therapeutic agents [8,11,12,13].

However, triptolide is a moderately lipophilic molecule with low *n*-octanol/water partition coefficient of 0.58 [14,15]. It does not partition well in melted lipid droplets during SLN preparation, representing a technological challenge to ensure satisfactory encapsulation efficiency (EE). Among the production methods of SLN, the microemulsion technique offers notable advantages such as ease of handling, a fast production process, and no need for employing special equipment. It was considered to be one of the most feasible methods for industrial production [16]. In addition, this technique was reported to successfully encapsulate hydrophilic compounds (which also tends to partition into the water phase during the production process) into SLN with high EE [17,18,19], indicating that microemulsion technique might play a beneficial role in circumventing the challenges encountered when formulating triptolide-loaded solid lipid nanoparticles (TP-SLN). Furthermore, experimental designs have been commonly used to simultaneously analyze the influence of different variables on the properties of drug delivery system [20], among them the central composite design (CCD) is suitable for pharmaceutical blending problems allowing optimization with the least number of experiments for selection of the best composition [21].

In this study, optimized TP-SLN was prepared on the basis of the predicted optimum levels of independent variables of CCD using the microemulsion technique. Stability in simulated gastric fluid and *in vitro* release profile of optimized TP-SLN were evaluated. In addition, it has been documented that reduced exposure of the drug to physiological constituents by encapsulation into the delivery system and slow drug release would decrease the toxicity profile of the drug [22,23]. We investigated if incorporation of TP into SLN could produce a protective effect on the gastric mucosa compared with the gastric irritation effect caused by a TP aqueous suspension.

## 2. Results and Discussion

### 2.1. Preparation of TP-SLN

TP-SLN was successfully prepared by the microemulsion technique. An oil-in-water microemulsion was spontaneously obtained as recognized by a clear solution after adding the heated water phase into the oil phase of the same temperature. Addition of a hot microemulsion to cold water led to precipitation of the lipid phase forming fine particles. High-temperature gradients facilitate rapid lipid crystallization and prevent lipid aggregation [8,12]. In a preliminary study, the critical variables including fraction of lipid (X_1_), surfactant to co-surfactant ratio (X_2_) and lipid to drug ratio (X_3_) that influenced the particle size, EE and DL of TP-SLN were selected, and single-factor experiments were performed to determine the appropriate ranges of these three variables.

### 2.2. Designing the Models

The observed particle size, EE and DL values from the 20 experiments are shown in Table 1. The statistical parameters computed by design-expert software indicated that quadratic polynomial model was the best fitted for the experimental data for all responses. The R^2^ values were 0.9585, 0.9467 and 0.9916 for Y_1_ (particle size), Y_2_ (EE) and Y_3_ (DL) models, respectively, which meant that the relationship between the variables and responses was well depicted by second order model. The quadratic polynomial equations in terms of coded levels for the three responses are as follows:

Particle size (nm) = 118.70 + 78.41X_1_ − 98.31X_2_ + 3.21X_3_ − 61.69X_1_X_2_ + 2.15X_1_X_3_ − 1.17X_2_X_3_ + 22.74X_1_^2^ + 60.75X_2_^2^ − 3.88X_3_^2^ (1)

Encapsulation efficiency (%) = 50.47 + 1.52X_1_ – 2 .65X_2_ − 2.31X_3_ − 0.27X_1_X_2_ + 0.23X_1_X_3_ + 1.08X_2_X_3_ − 2.30X_1_^2^ − 1.25X_2_^2^ − 0.30X_3_^2^ (2)

Drug loading (%) = 0.67 + 0.020X_1_ − 0.038X_2_ − 0.17X_3_ − 0.0029X_1_X_2_ − 0.0023X_1_X_3_ + 0.021X_2_X_3_ − 0.031X_1_^2^ − 0.017X_2_^2^ + 0.029X_3_^2^ (3)

in which X_1_, X_2_ and X_3_ were the descriptors for three independent variables.

The regression coefficients and analysis of variance (ANOVA) of the model parameters are listed in Table 2. A positive value in the regression equation represented an effect that favored the optimization due to synergism, while a negative value indicated an inverse relationship or antagonistic effect between the variables and the responses [24]. As can be seen in Table 2, fraction of lipid (X_1_) significantly increased the particle size of TP-SLN (*p* < 0.0001), yet increase in surfactant to co-surfactant ratio (X_2_) favored a smaller sized particle. EE was positively influenced by X_1_ but negatively influenced by X_2_ and X_3__,_ which also happened on DL though the intensity of the influence was lower. In addition, there were significant interactive parameters (*p* < 0.05) in particle size model which was the interaction between fraction of lipid and surfactant to co-surfactant ratio (X_1_X_2_); in EE and DL models which was the interaction between surfactant to co-surfactant ratio and lipid to drug ratio (X_2_ X_3_).

**Table 1:** The central composite design and resulting values of Y_1_ (particle size, nm), Y_2_ (encapsulation efficiency, %) and Y_3_ (drug loading, %). X_1_: fraction of lipid (%, w/w), X_2_: surfactant to co-surfactant ratio (w/w) and X_3_: lipid to drug ratio (w/w).

	Variables			Responses		
Experiments	X_1_	X_2_	X_3_	Y_1_	Y_2_	Y_3_
1	55.77	2.85	89.43	421	48.1	0.54
2	50.00	4.00	75.00	113.3	52.4	0.70
3	44.23	2.85	60.57	117.3	50.5	0.83
4	50.00	4.00	50.00	123.5	53.3	1.07
5	44.23	5.15	60.57	79.5	44.0	0.73
6	55.77	5.15	60.57	131.5	47.7	0.79
7	55.77	2.85	60.57	391.9	54.0	0.89
8	50.00	4.00	100.00	125.4	45.8	0.46
9	50.00	4.00	75.00	124.1	50.6	0.67
10	44.23	5.15	89.43	95.4	41.5	0.46
11	44.23	2.85	89.43	113.7	42.4	0.47
12	60.00	4.00	75.00	327.7	45.0	0.60
13	50.00	4.00	75.00	116.5	51.0	0.68
14	55.77	5.15	89.43	131.8	44.8	0.50
15	50.00	6.00	75.00	95.9	40.9	0.55
16	50.00	2.00	75.00	540.8	52.5	0.70
17	50.00	4.00	75.00	116.3	49.2	0.66
18	40.00	4.00	75.00	80.9	42.1	0.56
19	50.00	4.00	75.00	121.7	50.0	0.67
20	50.00	4.00	75.00	120.3	49.6	0.66

**Table 2:** Regression coefficients and analysis of variance (ANOVA) of the model parameters. X_1_: fraction of lipid (%, w/w), X_2_: surfactant to co-surfactant ratio (w/w) and X_3_: lipid to drug ratio (w/w).

Source	Particle size		Encapsulation efficiency		Drug loading	
	coefficient	*p*-value	coefficient	*p*-value	coefficient	*p*-value
Model		<0.0001		<0.0001		<0.0001
Intercept	118.70		50.47		0.67	
X_1_	78.41	<0.0001	1.52	0.0016	0.020	0.0029
X_2_	−98.31	<0.0001	−2.65	<0.0001	−0.038	<0.0001
X_3_	3.21	0.7522	−2.31	<0.0001	−0.17	<0.0001
X_1_ X_2_	−61.69	0.0008	−0.27	0.5713	−0.0029	0.6796
X_1_ X_3_	2.15	0.8730	0.23	0.6423	−0.0023	0.7484
X_2_ X_3_	−1.17	0.9305	1.08	0.0452	0.021	0.0140
X_1_^2^	22.74	0.0349	−2.30	<0.0001	−0.031	<0.0001
X_2_^2^	60.75	<0.0001	−1.25	0.0039	−0.017	0.0056
X_3_^2^	−3.88	0.6861	−0.30	0.3902	0.029	0.0001

### 2.3. Response Surface Analysis

The effect of the formulation variables on a response was assessed by studying the three-dimensional response surface plots. These plots were used to describe the interaction and quadratic effects of two independent variables on the response at one time, while keeping the third variable constant. 

#### 2.3.1. Effects on Particle Size

**Figure 1:**
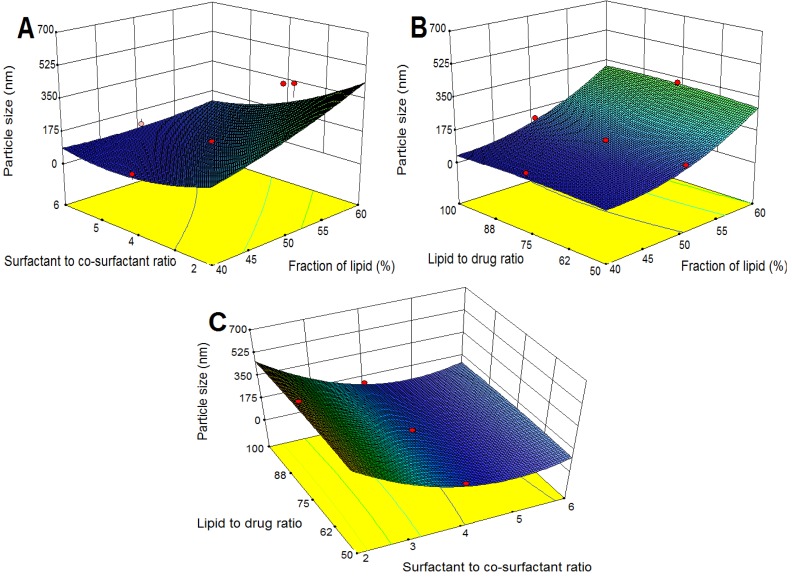
Response surface plots showing the effects of variables on particle size of TP-SLN. (**A**) fraction of lipid and surfactant to co-surfactant ratio, (**B**) fraction of lipid and lipid to drug ratio, and (**C**) surfactant to co-surfactant ratio and lipid to drug ratio.

The three-dimensional response surface plots for particle size are presented in Figure 1. As shown in Figure 1A,B, particle size increased with increasing fraction of lipid, which could be related to a viscosity increase in the dispersion, leading to higher surface tension and thus larger particle size [25]. Furthermore, increasing the particle size as a result of higher content of lipid might occur due to increased collision and aggregation of the nanoparticles [26], or relatively lack of enough surfactant for covering the surface of the particles [18]. However, as indicated in Table 2, X_2_ (surfactant to co-surfactant ratio) showed negative effect on particle size and by increasing X_2_, particle size decreased (Figure 1A,C), which suggested that the presence of a higher content of surfactant (Cremophor RH40) reduced interfacial tension more effectively and the lipid could become more homogenized in the aqueous phase and this caused the formation of smaller particles.

#### 2.3.2. Effects on Encapsulation Efficiency (EE)

**Figure 2:**
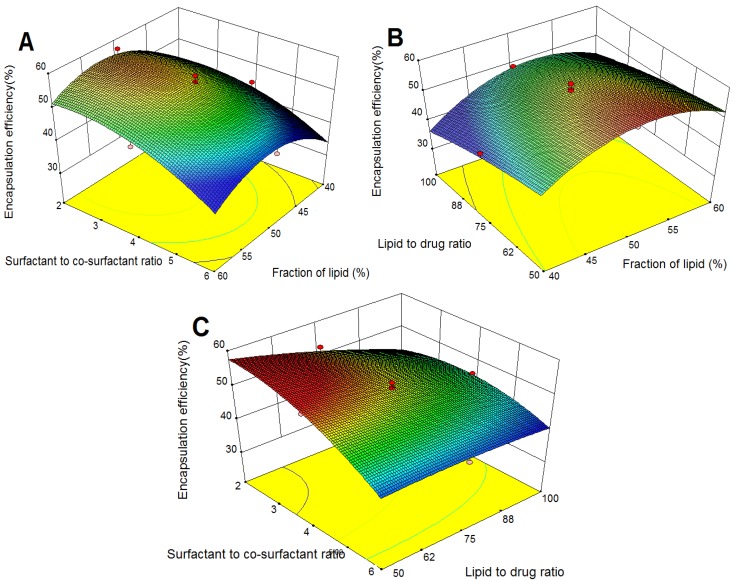
Response surface plots showing the effects of variables on EE of TP-SLN. (**A**) fraction of lipid and surfactant to co-surfactant ratio, (**B**) fraction of lipid and lipid to drug ratio, and (**C**) surfactant to co-surfactant ratio and lipid to drug ratio.

Figure 2A–C illustrate the response surface model for EE in response to the investigated variables. As shown in Figure 2A,B, EE improved with increases in fraction of lipid to an optimal maximum value. The possible reason could be that higher content of lipid afforded more space to accommodate the drug [21]. On the other hand, surfactant was needed to solubilize TP in the lipid (or allow TP to disperse in its coating) because of the low lipophilicity of TP. Consequently, EE gradually increased to its maximum point and then the relatively decreased content of surfactant might fail to load more TP. Lipid to drug ratio negatively influenced EE (Table 2). However, the intensity of the influence was relatively low (Figure 2B,C).

A significant negative effect of surfactant to co-surfactant ratio (X_2_) on EE was observed (Figure 2A,C). As the amount of surfactant increased, the surface of the formed SLN failed to absorb all surfactant molecules, which would result in the formation of micellar solution causing increased partition of TP from the SLN into water phase [20]. On the other hand, as reported in the literature, molecules with low lipophilicity could disperse or dissolve in the coating of lecithin [27], and a strong binding of phospholipids to the solid lipid matrix might occur to immobilize the interfacial film [28]. This immobilization lowered the partition of TP across the lecithin barrier to water phase. Therefore, lecithin (co-surfactant) decreased with increasing X_2_, which also led to decreased EE.

#### 2.3.3. Effects on Drug Loading (DL)

**Figure 3:**
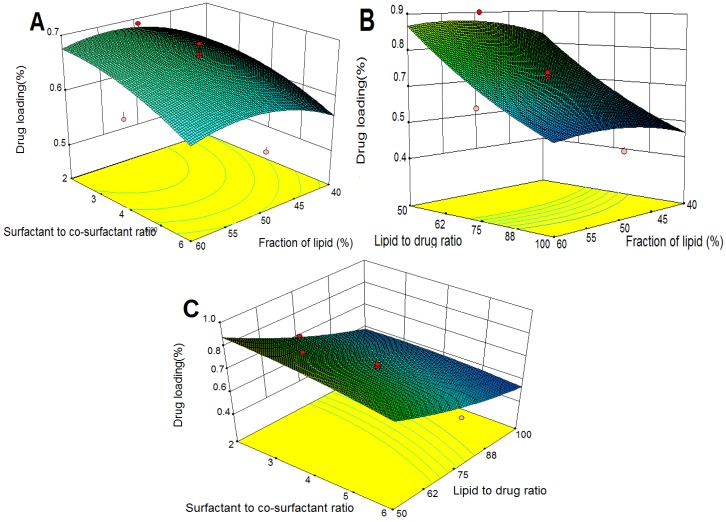
Response surface plots showing the effects of variables on DL of TP-SLN. (**A**) fraction of lipid and surfactant to co-surfactant ratio, (**B**) fraction of lipid and lipid to drug ratio, and (**C**) surfactant to co-surfactant ratio and lipid to drug ratio.

The three-dimensional response surface plots for DL are presented in Figure 3. As shown in Figure 3A,B, DL improved with increases in fraction of lipid (X_1_) to an optimal maximum value, and surfactant to co-surfactant ratio (X_2_) had a significantly negative effect on DL (Figure 3A,C), which suggested that X_1_ and X_2_ had similar effects on DL and EE. In addition, increased lipid to drug ratio significantly decreased DL (Figure 3B,C), which might due to the increase of lipid content and reduction of EE (Figure 2B,C).

### 2.4. Optimization and Validation

After analyzing the polynomial equations depicting the independent variables and responses, the formulation was optimized targeting the prescriptive criteria of low particle size, maximum EE and DL. The composition of optimum formulation was predicted as: level of fraction of lipid of 49.73%, surfactant to co-surfactant ratio of 3.25, and lipid to drug ratio of 55.27. The predicted values of particle size, EE and DL were 189.6 nm, 55.1% and 1.10%, respectively. The observed optimized formulation had particle size of (179.8 ± 5.7) nm, EE of (56.5 ± 0.18)% and DL of (1.02 ± 0.003)%, which were in good agreement with the predicted values.

### 2.5. *In Vitro* Release

**Figure 4:**
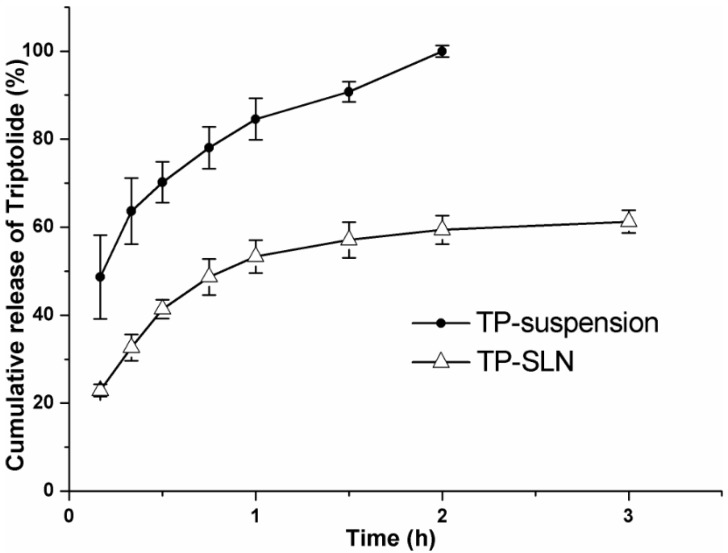
*In vitro* release profile of TP-suspension and optimized TP-SLN. 0.1 M HCl (pH 1.2) containing 10% (v/v) ethanol was selected as the release medium and the experiment was performed by the dialysis bag diffusion technique at 37 °C. Results are expressed as mean ± SD (n = 3).

Figure 4 shows the cumulative release of drug from control TP-suspension and optimized TP-SLN. Complete release (100%) was achieved within 2 h from TP-suspension, which indicated rapid diffusion of TP. This confirmed that a sink condition was accomplished and that the dialysis membrane used did not limit drug release [29]. In contrast, the release of TP from TP-SLN was about 1.68-fold slower than that from the suspension (59.4%). In addition, TP showed 53.3% of drug release within the first hour followed by sustained release from TP-SLN. The presence of the free TP in the external phase and on the surface of the nanoparticles might be the reason for the burst release [30]. Because of the solid matrix of the SLN and the subsequent drug immobilization [13], a slow and sustained release profile would therefore be expected.

### 2.6. Stability Study in Simulated Gastric Fluid

**Figure 5:**
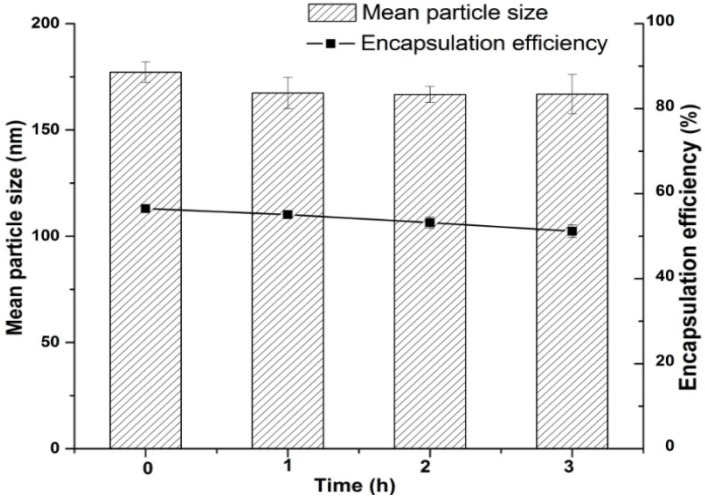
Stability study of optimized TP-SLN in simulated gastric fluid. Results are expressed as mean ± SD (n = 3).

The results of the stability of optimized TP-SLN in simulated gastric fluids are shown in Figure 5, which revealed a slight but insignificant decrease in the particle size (*p* > 0.05). EE of TP-SLN was measured during 3 h of incubation in gastric medium and a release of 9.3% of the initial amount of encapsulated TP was detected. These changes might be attributed to the protective coating of Cremophor RH40 (polyoxyl 40 hydrogenated castor oil) on the surface of SLN, it could protect Compritol 888 ATO (glyceryl behenate) and Lipoid E 80 (egg lecithin) from the acidic environment and against enzyme degradation, but some TP could still diffuse from the shell of SLN [31,32]. It is worth mentioning that the majority of the drug (91%) was protected.

### 2.7. Assessment of Gastric Mucosa Irritation

**Figure 6:**
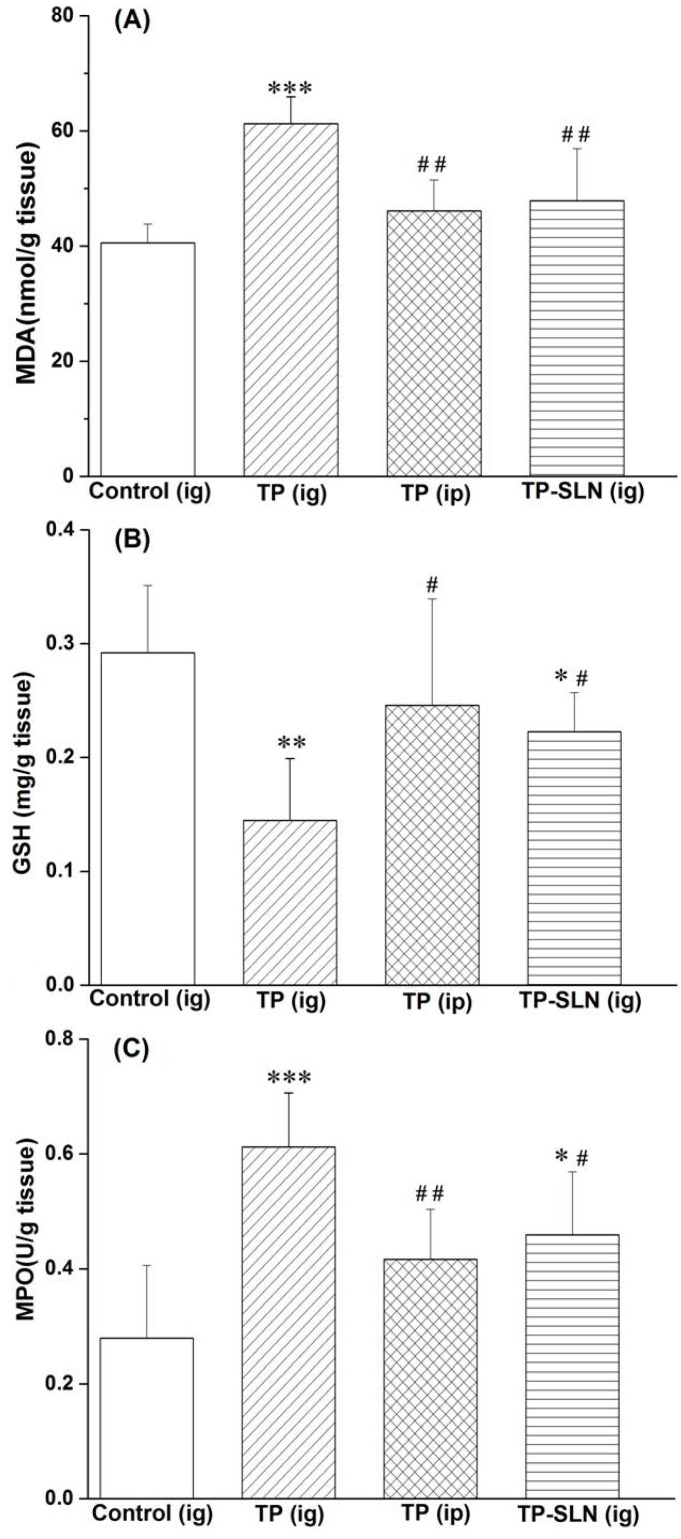
Levels of malonyldialdehyde (MDA) and glutathione (GSH), and activity of myeloperoxidase (MPO) in gastric mucosa after administration of TP and TP-SLN in rats. The results are expressed as mean ± SD (n = 6). * *p* < 0.05, ** *p* < 0.01, *** *p* < 0.001 compared with control group; # *p* < 0.05, ## *p* < 0.01 compared with TP (ig) group. Notes: ig, intragastric; ip, intraperitoneal injection.

It is well recognized that oxidative stress mediates cellular injury in the gastrointestinal tract [33,34,35], and that glutathione (GSH) is one of the important components presenting in high concentration in the gastric tissues, depletion of GSH in gastric mucosa can result in lipid peroxidation and gastric damage [36]. As shown in Figure 6A,B, there were significant increase in malonyldialdehyde (MDA, *p* < 0.001) and reduction in GSH (*p* < 0.01) in the TP (ig)-treated group compared with the control group. However, when TP was administrated by intraperitoneal injection, no obvious changes of MDA and GSH were observed. On the other hand, SLN significantly reduced the lipid peroxidation levels induced by oral administration of TP. Myeloperoxidase (MPO) was measured as a maker of neutrophil infiltration into the gastric mucosal tissues [37,38,39]. As shown in Figure 6C, TP significantly increased (*p* < 0.001) the activity of myeloperoxidase after administration by intragastric route (but not after intraperitoneal injection). In contrast, the oral administration of TP-SLN significantly reduced (*p* < 0.05) MPO activity compared with the TP (ig)-treated group.

**Figure 7:**
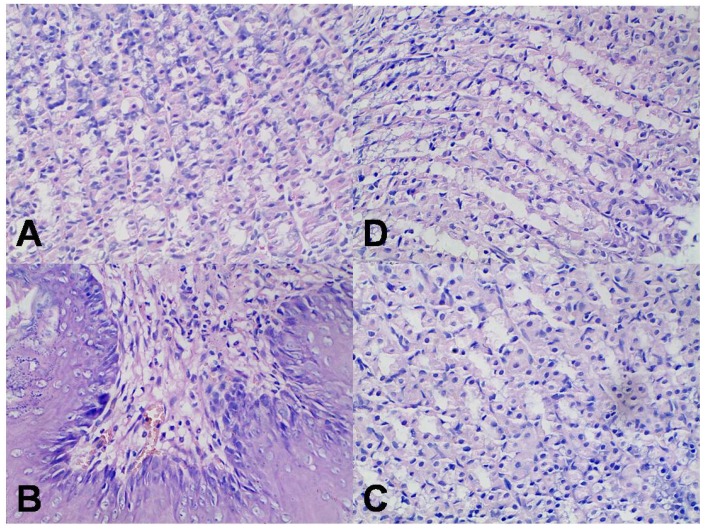
Histological examination of gastric tissue of rats (hematoxylin and eosin staining, 400 ×). Results were derived from oral administration of TP (**B**) and TP-SLN (**C**), intraperitoneal injection of TP (**D**) at the dose of 1.0 mg/kg. Control group (**A**): oral administration of 0.5% sodium carboxymethyl cellulose solution.

When TP-suspension was administered orally to rats, marked hyperemia was observed on the gastric mucosal surface, while in other groups, hardly any hyperemia was observed. The protective effect of loading TP into SLN was further confirmed by histological examination. For the group given an oral dose of TP-suspension at 1.0 mg/kg, infiltration of inflammatory cells was clearly seen in the gastric mucosa of the rats (Figure 7B). However, for the TP intraperitoneal injection group and TP-SLN oral group, there was hardly any evidence of the gastric mucosa irritation (Figure 7C,D).

The above results suggested that SLN possessed a reducing effect against irritation in rat stomach tissues induced by TP. This could be attributed to the encapsulation of TP into SLN, which reduced the direct contact of drugs with the mucosal surface. Our findings are consistent with previous observations which showed that irritation to tissues induced by some drugs could be minimized by SLN encapsulation [40,41,42]. Furthermore, due to their small particle size, lipid nanoparticles exhibited bioadhesion to the gastric tract wall thereby achieving a longer retention time [43], releasing TP very gradually, which reduced local high concentration. In addition, free TP in SLN dispersion might dissolve in the gastric fluid in the form of solubilized surfactant micelles and was adsorbed in molecular form, so that no large crystals came into contact with the stomach mucosa causing irritation [6].

## 3. Experimental

### 3.1. Materials

Triptolide was purchased from Nanjing Zelang Medical Technology Co., Ltd (Nanjing, China, purity >98% by HPLC). Pepsin was supplied by Sinopharm Chemical Reagent Co., Ltd (Beijing, China). Cremophor RH40 (polyoxyl 40 hydrogenated castor oil) was obtained from BASF (Ludwigshafen, Germany). Compritol 888 ATO (glyceryl behenate) and Transcutol HP (diethylene glycol monoethyl ether) were from Gattefossé SAS (Saint Priest Cedex, France). Lipoid E 80 (egg lecithin) was supplied by Lipoid GmbH (Ludwigshafen, Germany). Solvents were of HPLC grade and water was obtained from a Milli-Q water purification system (Millipore, Bedford, MA, USA). Other chemicals were of analytical grade.

### 3.2. Animals

The protocol of this study was approved by the Ethical Committee of Huazhong University of Science and Technology. Male Sprague-Dawley rats (180–200 g) were purchased from Laboratory Animals Center of Tongji Medical College of Huazhong University of Science and Technology (Wuhan, China). They were housed in an air conditioned room under a 12 h light/dark cycle with free access to food and water. They were acclimatized for one week before the experiments.

### 3.3. Preparation of TP-SLN Using the Microemulsion Technique

TP-SLN was prepared using the microemulsion technique [17]. Briefly, drug (TP), solid lipid (Compritol 888 ATO), surfactant (Cremophor RH40) and co-surfactant (Lipoid E 80 dissolved in Transcutol HP at a ratio of 1:1, w/w) were mixed and heated together at 85 °C under magnetic stirring, then water (85 °C) was added to obtain an optically transparent microemulsion. This hot microemulsion was dispersed into cold water (2~4 °C) under vigorous stirring using a ratio of 1:5, and subsequently filtered through 1 μm membrane filters. The final aqueous dispersion of TP-SLN was then obtained and stored at 4 °C.

### 3.4. Central Composite Design (CCD)

The critical independent variables (fraction of lipid (X_1_), surfactant to co-surfactant ratio (X_2_) and lipid to drug ratio (X_3_)) influencing the properties of the produced TP-SLN were selected, and a three-factor, five-level CCD was developed. Particle size (Y_1_), EE (Y_2_) and DL (Y_3_) were selected as responses. The coded and actual values of the variables are given in Table 3. According to the CCD, a set of 20 experiments was designed including eight factorial points, six axial points and six replicated central points.

**Table 3:** Independent variables and their levels of CCD.

**Independent Variables**	**Coded levels**				
	−1.732	−1	0	+1	+1.732
**X_1_:** **fraction of lipid** **(%, w/w)**	40.00	44.23	50.00	55.77	60.00
**X_2_:** **surfactant to co-surfactant ratio (w/w)**	2.00	2.85	4.00	5.15	6.00
**X_3_:** **lipid to drug ratio** **(w/w)**	50.00	60.57	75	89.43	100.00

Statistical analysis was performed by Design-Expert^®^ 8.0 software (Stat-Ease Inc., Minneapolis, MN, USA). The best fitting mathematical model was chosen by comparing several statistical parameters of various polynomial models consisting of the multiple correlation coefficient (R^2^), the adjusted multiple correlation coefficient (adjusted R^2^), the predicted multiple correlation coefficient (predicted R^2^) and lack-of-fit [44]. Analysis of variance (ANOVA) was employed to assess the significance of differences. The *p*-values < 0.05 were considered to be statistically significant. In order to achieve a better understanding of the independent variables influence on responses, response surface plots of the fitted models were constituted also using Design-Expert^®^ 8.0 software. The optimized formulation was prepared for further evaluation.

### 3.5. Characterization of TP-SLN

#### 3.5.1. Particle Size

The mean particle size was measured by photon correlation spectroscopy (Nano-ZS90 zetasizer, Malvern Instruments Corp., Worcestershire, UK) at 25 °C with a 90° scattering angle. Milli-Q water was used as a dispersant medium.

#### 3.5.2. Encapsulation Efficiency (EE) and Drug Loading (DL)

Triptolide was analyzed using Agilent 1100 HPLC (Agilent Technologies, Palo Alto, CA, USA). Analyses were performed at 25 °C using a ZORBAX Eclipse Plus C_18_ column (250 mm × 4.6 mm, 5 μm) with a Agilent guard cartridge. The mobile phase consisted of methanol: water (45:55, v/v). The flow rate and detection wavelength were 1.0 mL/min and 218 nm, respectively.

Free TP was separated by subjecting 0.5 mL of freshly prepared TP-SLN to ultrafiltration (Amicon ultra, Millipore Co., Bedford, MA, USA, molecular weight cutoff 10 kDa) for 40 min at 4,000 g at 4 °C. Filtrate containing the free drug was taken for HPLC analysis. Total amount of drug was determined by disrupting TP-SLN dispersion (0.5 mL) with a mixed solvent composed of dichloromethane and methanol (1:10) followed by vortex for 5 min. After filtration through a 0.45 μm membrane filter, the resulting solution was also analyzed by HPLC. EE and DL were calculated using the equation given below [45]:

EE (%) = (W_Total_－W_Free_)/W_Total_ × 100 (4)

DL (%) = (W_Total_ – W_Free_)/W_Lipid_ × 100 (5)

in which W_Total_, W_Free_ and W_Lipid_ were the weights of total drug and the unencapsulated drug, and the lipid added in system, respectively.

### 3.6. *In Vitro* Release

*In vitro* release was performed in 0.1 M HCL (pH 1.2) containing 10% (v/v) ethanol by the dialysis bag diffusion technique [46]. The bag (molecular weight cutoff 8~14 kDa) was soaked in water for 12 h prior to experiment. The optimized TP-SLN or TP-suspension (TP dispersed in 0.5% sodium carboxymethyl cellulose solution) were placed inside the bag (equivalent to 0.5 mg TP), tied at both the ends and dipped into 50 mL medium in a conical flask. Then conical flask was put into an incubator shaker (CIMO, Shanghai, China). Shaking was maintained at 100 rpm at 37 °C. Aliquots (0.5 mL) were withdrawn at pre-set time intervals (0.17, 0.33, 0.50, 0.75, 1, 1.5, 2 and 3 h) and immediately replaced by an equal volume of fresh release medium. Amount of released drug was also determined by the HPLC method mentioned above. A profile showing the cumulative drug release as a function of time was plotted.

### 3.7. Stability Study in Simulated Gastric Fluid

The simulated gastric fluid (SGF) was constituted with sodium chloride (0.2 g), hydrochloric acid (0.7 mL) and pepsin (0.32 g) in 100 mL of water [47]. The optimized TP-SLN was diluted at a final concentration of 10% (v/v) and incubated at 37 °C in SGF. Samples were collected at times 0, 1, 2 and 3 h, and then evaluated for changes in particle size and EE.

### 3.8. Assessment of Gastric Mucosa Irritation

Twenty-four SD rats were divided into four groups with six rats each and fasted for 24 h but with free access to water. One group was given TP (aqueous solution of 20% propylene glycol) via intraperitoneal injection. The other three groups were orally administered with 0.5% sodium carboxymethyl cellulose solution (control), TP-suspension and optimized TP-SLN, respectively. Dose of TP was 1.0 mg/kg. One hour after administration, all rats were anesthetized and sacrificed. Their stomachs were removed and rinsed thoroughly with physiological saline. The stomachs were opened along the line of greater curvature and then spread flat and examined for signs of injury macroscopically. Afterward, gastric tissue samples were fixed overnight in 4% paraformaldehyde and processed for paraffin sectioning followed by hematoxylin and eosin (H&E) staining for microscopic examination. The gastric mucosa was scraped with glass slides for measurement of biochemical parameters [34,48] including malonyldialdehyde (MDA), glutathione (GSH) and myeloperoxidase (MPO). These parameters were determined using reagent kits (Nanjing Jiancheng Biotechnology Institute, Nanjing, China) according to the manufacturer’s protocols. The statistical analysis of differences between groups was performed using Student’s t-test. Results were presented as mean ± standard deviation (SD). *p* < 0.05 was considered to be significant.

## 4. Conclusions

In the present study, we report incorporation of TP into SLN by the microemulsion technique. Central composite design (CCD) was employed to evaluate the effect of the formulation variables on particle size, encapsulation efficiency (EE) and drug loading (DL). The optimized TP-SLN offered benefits in terms of relatively high EE, good stability in simulated gastric fluid, and prolonged release profile *in vitro*. In addition, SLN had a potential of preventing gastric mucosa irritation caused by oral administration of TP in rats, this could be attributed to reduced lipid peroxidation levels and inflammation of the stomach mucosa.
